# Efficacy and toxicity of re-irradiation for esophageal cancer patients with locoregional recurrence: a retrospective analysis

**DOI:** 10.1186/s13014-020-01685-2

**Published:** 2020-10-21

**Authors:** Kaikai Zhao, Youjiao Si, Liangchao Sun, Xiangjiao Meng, Jinming Yu

**Affiliations:** 1 Department of Radiation Oncology, Yantai Affiliated Hospital of Binzhou Medical University, Yantai, 264100 China; 2 Department of Radiology, Yantai Affiliated Hospital of Binzhou Medical University, Yantai, 264100 China; 3 Department of Radiation Oncology, Shandong Cancer Hospital and Institute, Shandong First Medical University and Shandong Academy of Medical Sciences, Jinan, 250117 China

**Keywords:** Esophageal squamous cell carcinoma, Locoregional recurrence, Re-irradiation, Prognosis

## Abstract

**Introduction:**

There is no standard treatment for locoregional recurrent (LR) esophageal squamous cell carcinoma (ESCC) patients treated with radiotherapy (RT) previously. This retrospective study aimed to examine the efficacy and toxicity of re-irradiation (re-RT) for ESCC patients with LR.

**Patients and methods:**

A total of 252 patients were enrolled. Gross tumor volumes for re-RT were defined using contrast enhanced computed tomography and/or positron emission tomography/computed tomography. Overall survival (OS), after recurrence survival (ARS) and toxicities were assessed.

**Results:**

Through a median follow-up of 38 months, the median OS and ARS were 39.0 and 13.0 months, respectively. The 6-, 12-, and 24-month ARS rates were 81.9%, 50.5%, and 21.8%, respectively. Multivariate analyses showed that chemotherapy, esophageal stenosis and recurrence-free interval (RFI) may be independent prognostic factors for ARS. The incidence of esophageal fistula/perforation (EP), radiation-induced pneumonitis and esophagorrhagia was 21.4%, 12.8% and 9.1%, respectively. RFI ≤ 12 months, esophageal stenosis and fat space between tumor and adjacent tissue disappeared were independent risk factors for the development of EP after re-RT.

**Conclusions:**

Re-RT was feasible for LR ESCC patients after RT initially, the complication occurred in re-RT is acceptable. Patients with RFI ≤ 12 months, esophageal stenosis and fat space between tumor and adjacent tissue disappeared should be closely observed during and after re-RT.

## Introduction

Esophageal cancer is the 6th most common cause of cancer deaths worldwide because of its high malignant potential and poor prognosis [1]. Locoregional recurrence (LR) is the major type of failure form in 24–50% of the patients after initial therapy such as surgery and/or chemoradiotherapy (CRT) [2, 3], and in-field relapse after radiotherapy (RT) occurred in more than 20% of patients [4, 5]. The 5-year survival rate drops dramatically down to 0–11% once recurrence occurs [6, 7].

Patients with good physical conditions need to be given active treatment to local recurrent disease to achieve better survival. There is no standard treatment for patients with LR so far. Surgery plays an important role in achieving locoregional control in patients with LR esophageal carcinoma [8] but salvage esophagectomy may cause serious surgery-related complication and hospital mortality. CRT has curative potential for LR esophageal squamous cell carcinoma (ESCC) patients, but the clinical benefit and safety is not demonstrated very well due to the small number of cases [9–17]. In this study, we retrospectively analyzed the efficacy and toxicity of re-irradiation (re-RT) in patients with LR after radical radiotherapy or postoperative adjuvant radiotherapy on a relative large sample.

## Materials and methods

### Patients

All of the 252 ESCC patients were confirmed by pathology, collected from our hospital from January 2000 to December 2018. Patients were selected meeting the following criteria: (1) primary ESCC was pathological confirmed, (2) histological and/or positron emission tomography/computed tomography confirmation of LR including primary failure (PF), regional lymph node recurrence (LN) or PF combined LN relapsed in-field after initial RT; (3) no evidence of esophageal perforation or ulcer. The exclusion criteria were as follows: (1) history of other malignancies, (2) distant metastases.

Clinical or pathological stage was done according to the 7th edition of the American Joint Committee on Cancer TNM staging system. Toxicities were evaluated according to the National Cancer Institute Common Toxicity Criteria version 3.0. The current study was approved by the Ethics Committee of our hospital.

### Treatment

RT was delivered via a 6 MV X-ray linear accelerator, the total doses of primary RT and re-RT are listed in Tables 1 and 2. For the re-RT, the gross tumor volume (GTV) after recurrence was defined as the region of recurrence determined by contrast enhanced computed tomography and/or positron emission tomography/computed tomography. The clinical target volume (CTV) was defined as the GTV plus a margin of 1.0–2.0 cm on each side. The planning target volume (PTV) for re-RT was defined as the CTV or GTV plus a 0.5–1.0 cm margin in all directions, according to previous RT techniques and exposure dose to organs at risk. Partially or all the re-RT target volumes were in the initial radiation fields. The biological effectiveness of radiation schedule was calculated by the biologically effective dose (BED) formula: BED = n × d (1 + d/(α/β)), α/β = 10 [18]. The total dose to the spinal cord was limited not to exceed the maximal dose of 45 Gy except for a few patients, considering the time interval between primary RT and re-RT. V_20_ were limited within 30% in the first RT and less than 20% during re-RT for the total lungs.

**Table 1:** Characteristics of 252 patients with locoregional recurrent ESCC at initial treatment

Variables	Number	Percent
Age (years)		
> 60	126	50.0
≤ 60	126	50.0
Gender		
Male	206	81.7
Female	46	18.3
Smoking		
Yes	122	48.4
No	130	51.6
Alcohol consumption		
Yes	108	42.9
No	144	57.1
Family history of malignancy		
Yes	45	17.9
No	207	82.1
Primary tumor location		
Upper thoracic	72	28.6
Middle and lower thoracic	180	71.4
Length (cm)		
≤ 4	152	60.3
4 < to ≤ 6	71	28.2
> 6	29	11.5
Macroscopic tumor type		
Medullary	134	53.2
Ulcerative	83	32.9
Constrictive	15	6.0
Mushroom	20	7.9
Tumor differentiation		
Higher	36	14.3
Middle	143	56.7
Lower	73	29.0
T stage		
T1–T2	79	31.3
T3–T4	173	68.7
N stage		
N0	110	43.7
N1	111	44.0
N2	31	12.3
Initial clinical/pathological stage		
I–II	117	46.4
III–Iva	135	53.6
Radiation dose (Gy)		
≤ 50	64	25.4
> 50	188	74.6
Daily dose (Gy)		
1.8	61	24.2
2.0	180	71.4
> 2.0	11	4.4
Initial treatment		
CRT	167	66.3
Surgery + CRT	85	33.7
Radiotherapy technique		
Conventional treatment	20	7.9
3D-CRT	113	44.9
IMRT	119	47.2

**Table 2:** Characteristics of 252 patients with locoregional recurrent ESCC at re-RT

Variables	Number	Percent
Age (years)		
> 60	167	66.3
≤ 60	85	33.7
Pattern of recurrence		
Regional lymph node recurrence only	108	42.9
Local failure	96	38.1
Both	48	19.0
Recurrence-free interval (months)		
≤ 12	76	30.2
> 12	176	69.8
Chemotherapy		
Yes	198	78.6
No	54	21.4
Radiation dose (Gy), BED_10_		
≤ 60	133	52.8
> 60	119	47.2
Daily dose (Gy)		
1.15–1.5 bid	48	19.0
1.8	60	23.8
2	144	57.1
Radiotherapy technique		
3D-CRT	40	15.9
IMRT	212	84.1

All patients received chemotherapy (CT) at the initial treatment, which was treated with 2 to 6 courses CT (median 4). CT regimens were mainly as 5-fluorouracil or paclitaxel plus cisplatin. 198 (78.6%) patients received CT combined with re-RT. The CT regimen was basically the same as before, and the number of cycles of CT was 1–6 (median 2).

### Follow-up

Overall survival (OS) time was defined as from the time of the initial treatment to death or the time of last follow-up. The after-recurrence survival (ARS) time was calculated from the date of relapse to the date of death or last follow-up. The recurrence-free interval (RFI) was defined as the time of interval from the end of initial treatment to the recurrence diagnosis [17]. The degree of esophageal stenosis was evaluated with esophagography by the method described before [19] and level ≥ 2 was defined as esophageal stenosis in this study. Patients were considered censored if without end events at the end of the study. Esophageal fistula/perforation (EP), radiation pneumonitis (RP) and esophagorrhagia were recorded.

### Statistical analysis

All statistical tests were conducted by using the SPSS Statistics version 22.0 (IBM Corporation, Armonk, NY, USA) and all figures were produced using GraphPad Prism 8.0 (Graphpad Software, USA). *p *value < 0.05 was considered statistically significant. The rates of survival curves were calculated using the Kaplan–Meier analysis method and log-rank tests. The Cox regression model was employed for the univariate analysis and multivariate analysis. The risk factors associated with the development of EP were analyzed by the forward logistic regression method.

## Results

### Patient characteristics

At initial treatment, 167 patients received radical RT and 85 patients received RT after surgery. The characteristics of the tumors and cohort are summarized according to the initial treatment (Table 1, 2). The median age was 66 years (range 39–88 years) at the time of diagnosis and 69 years (range 45–90 years) in the re-RT. The median RFI was 20 months (range 3–204 months). The median length of these lesions at initial diagnosis was 5.0 cm (range 1.5–13.5 cm). PF and LN were the most common failure pattern for radical RT (50.3%) and surgery (70.6%) respectively. The majority of patients (95.6%) received conventional fractionation treatment in the initial RT and 19.0% patients received hyperfractionation radiotherapy in re-RT. Among ESCC patients, the median initial radiation dose was 72 Gy (range 42.5–84.0 Gy), median re-RT dose was 60 Gy (range 12.0–86.3 Gy), and median total radiation dose (BED) was 131.5 Gy (range 84.0–158.3 Gy). 150 patients (59.5%) had Level 1, 65 patients (25.8%) had Level 2, 20 patients (7.9%) had Level 3, and 17 patients (6.8%) had Level 4 stenosis.

### OS and ARS

**Fig. 1:**
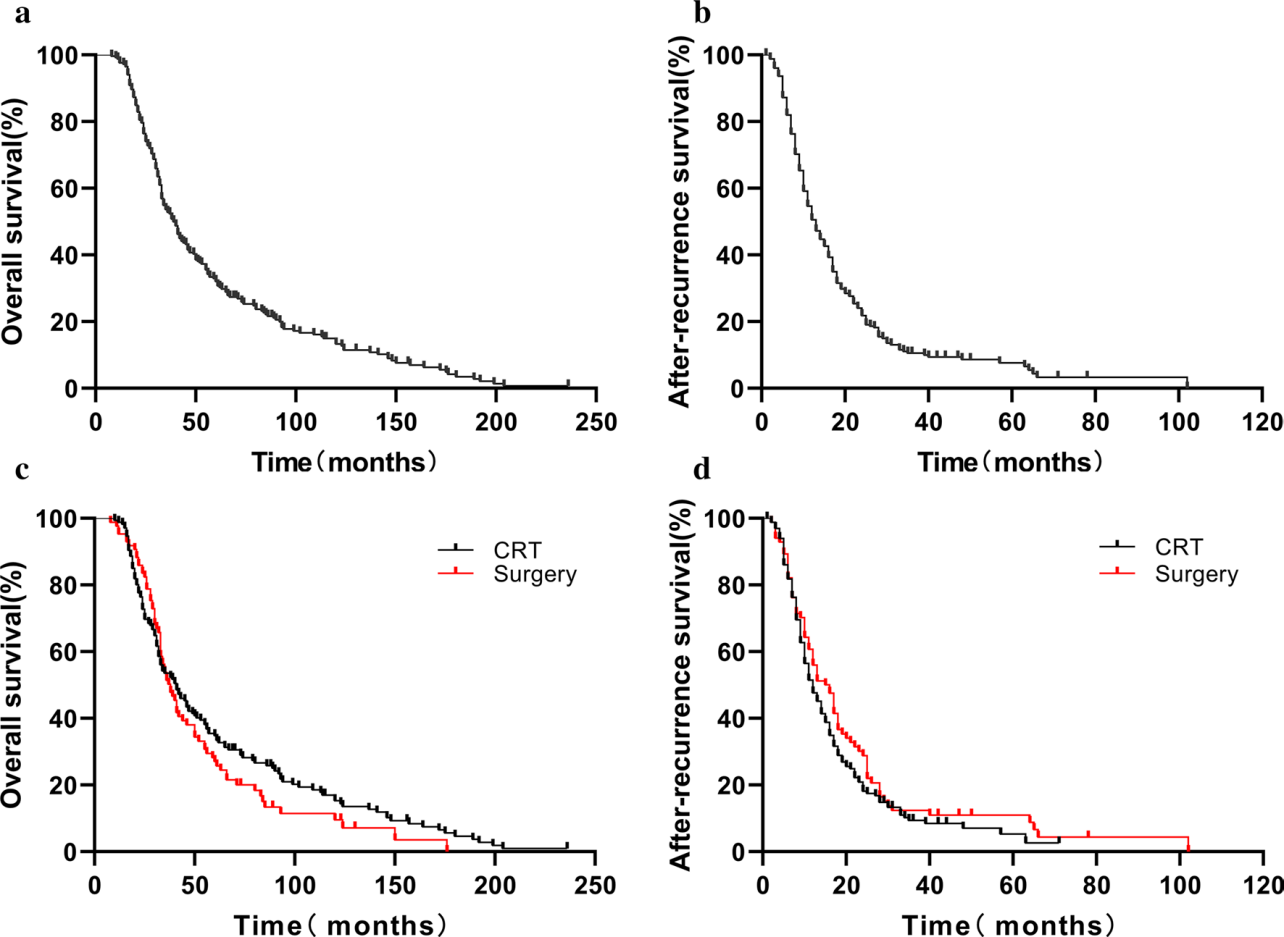
**a** Kaplan–Meier curve of OS for 252 patients with locoregional recurrent ESCC. **b** Kaplan–Meier curve of ARS for 252 patients with locoregional recurrent ESCC. **c** Kaplan–Meier curve of OS for 167 patients received RT initially and RT after surgery, respectively. **d** Kaplan–Meier curve of ARS for 85 patients received RT initially and RT after surgery

From initially diagnosed with ESCC, median follow-up was 38 months (range 8–236 months). 18 patients (7.1%) were still living, 224 patients (88.9%) had died including 1 patient died of suicide, 10 patients (4.0%) were lost to follow-up. The median OS of the 252 patients was 39.0 months (Fig. 1a) and the 1-, 3-, and 5-year OS rates were 97.6%, 52.8%, and 32.1%, respectively. The median ARS was 13.0 months (Fig. 1b) and the 6-, 12-, and 24-month ARS rates were 81.9%, 50.5%, and 21.8%, respectively. For the radical RT group, the median OS was 41.0 months (Fig. 1c) and the 1-, 3-, and 5-year OS rates were 98.8%, 53.6%, and 34.8%, respectively. The median ARS was 12.0 months (Fig. 1d) and the 6-, 12-, and 24-month ARS rates were 81.8%, 47.7%, and 18.1%, respectively. For the surgery group, the median OS was 38.0 months (Fig. 1c) and the 1-, 3-, and 5-year OS rates were 95.3%, 51.4%, and 27.0%, respectively. The median ARS of the 85 patients were 16.0 months (Fig. 1d) and the 6-, 12-, and 24-month ARS rates were 82.2%, 56.0%, and 28.8%, respectively.

### Prognostic factor analysis for ARS

**Fig. 2:**
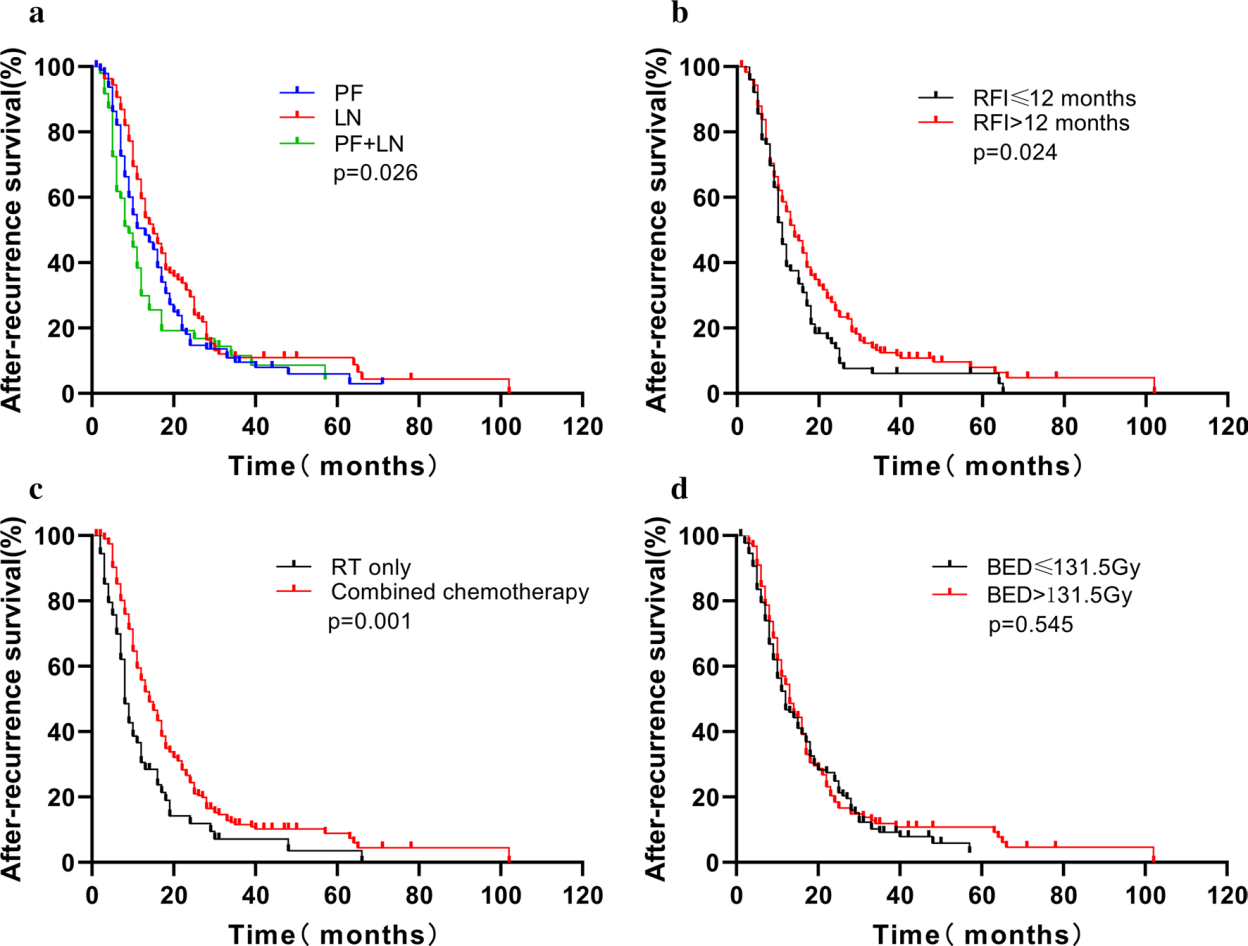
Kaplan–Meier survival curves. **a** Survival of patients who had PF, LN or PF combined with LN relapse. **b** Survival of patients who had an RFI ≤ 12 months versus RFI > 12 months. **c** Survival of patients who received RT only versus RT combined chemotherapy. **d** Survival of patients who received total RT dose > 131.5 Gy versus ≤ 131.5 Gy

We evaluated the relationship between ARS and clinicopathological features. In the univariate analysis (Table 3), median ARS was significantly longer for patients who occurred LN recurrence compared with those who occurred PF with/without LN recurrence (15.0 vs. 9.0 vs. 13.0 months, *p* = 0.026) (Fig. 2a). The median ARS of patients with RFI time > 12 months was longer than the patients with RFI time ≤ 12 months (14 vs. 11 months, *p* = 0.024) (Fig. 2b). Re-RT combined with chemotherapy can improve ARS (14 vs. 8 months, *p* = 0.001) (Fig. 2c). The ARS was similar for patients received a total radiation BED > 131.5 Gy compared with those for patients received an total radiation dose ≤ 131.5 Gy (*p* = 0.545) (Fig. 2d). Multivariate factor analysis for ARS revealed that CT, esophageal stenosis and RFI time may be independent prognostic factors for ARS (Table 4).

**Table 3:** Prognostic factors for ARS by univariate analysis

Variable	No. of patients	Survival rate, %			MST, mo (95% CI)	*p* Value
		6-m	12-m	24-m		
Sex						0.795
Male	206	81.8	50.8	21.8	12 (10.250–13.750)	
Female	46	82.6	49.1	21.8	11 (7.202–14.798)	
Re-RT age						0.316
≤ 60	85	83.3	54.8	26.9	16 (12.706–19.294)	
> 60	167	81.2	48.3	19.1	12 (9.958–14.042)	
Alcohol abuse						0.687
Yes	108	82.2	48.2	19.3	12 (9.679–14.321)	
No	144	81.7	52.3	23.8	13 (10.172–15.828)	
Smoking						0.683
Yes	122	81.0	48.2	19.9	12 (10.182–13.872)	
No	130	82.8	52.7	23.6	13 (9.675–16.325)	
Primary tumor location						0.773
Upper	72	83.3	51.4	17.2	13 (8.857–17.143)	
Middle/lower	180	81.3	50.1	23.6	13 (11.044–14.956)	
Length (cm)						0.573
≤ 4	152	86.0	51.5	23.2	13 (10.043–15.957)	
> 4 to ≤ 6	71	76.1	45.7	16.4	12 (9.349–14.651)	
> 6	29	75.0	51.7	27.3	14 (10.111–17.889)	
Tumor differentiation (n, %)						0.647
Higher	36	77.1	51.4	31.3	17 (9.200–24.800)	
Middle	143	84.4	49.7	18.3	12 (10.019–13.981)	
Lower	73	79.4	51.5	23.7	13 (9.684–16.316)	
Failure patterns						0.026
Primary failure	93	82.1	50.5	14.7	13 (9.216–16.784)	
Regional lymph node recurrence	106	90.6	59.5	29.5	15 (12.118–17.882)	
Combined	53	61.8	29.8	11.5	9 (6.127–11.873)	
Initial treatment						0.173
CRT	167	82.2	56.0	28.8	16 (12.311–19.689)	
Surgery	85	81.8	47.7	18.8	12 (9.960–14.040)	
Initial stage						0.966
I–II	117	83.6	51.7	19.4	14 (11.386–16.614)	
III–Iva	135	80.4	49.4	23.7	12 (9.942–14.058)	
RFI time						0.024
≤ 12 months	76	77.6	38.9	13.8	11 (9.600–12.400)	
> 12 months	176	83.8	55.6	25.4	14 (11.622–16.378)	
Chemotherapy						0.001
Yes	198	85.2	55.8	24.4	14 (11.739–16.261)	
No	54	94.4	30.5	11.9	8 (6.602–9.398)	
Salvage radiation dose (BED_10_)						0.326
≤ 60 Gy	133	80.9	45.0	23	12 (10.440–13.560)	
> 60 Gy	119	83.1	56.6	20.7	14 (11.791–16.209)	
Total radiation dose (BED_10_)						0.545
≤ 131.5 Gy	130	79.5	46.7	24.9	12 (9.115–14.885)	
> 131.5 Gy	122	84.4	54.5	18.5	13 (10.159–15.841)	
Esophageal stenosis						0.070
Yes	102	82.0	50.4	14.6	13 (10.599–15.401)	
No	150	81.9	50.6	26.5	13 (10.200–15.800)	
Pain in the chest or/and back						0.761
Yes	25	80.0	42.5	18.9	11 (6.314–15.686)	
No	227	82.1	51.4	22.1	13 (11.117–14.883)	
Fat space between tumor and adjacent tissue disappeared						0.121
Yes	64	82.6	46.1	12.9	12 (9.416–14.584)	
No	188	81.7	52.1	24.7	13 (10.394–15.606)	
BMI						0.269
≤ 20	74	82.2	46.0	17	12 (9.745–14.255)	
> 20	178	81.8	52.4	23.8	14 (11.483–16.517)	
Hemoglobin (g/L)						0.159
≤ 12	95	80.6	46.8	18.7	12 (9.446–15.554)	
> 12	157	82.7	52.7	23.7	13 (10.200–15.800)	
Albumin (g/L)						0.281
≤ 40	87	83.7	48.0	17.9	12 (10.095–13.905)	
> 40	165	80.9	51.8	23.8	13 (10.195–15.805)	

*MST* median survival time, *CI* confidence interval

**Table 4:** Multivariate Cox analysis of the ARS for re-RT

Variable	*p* Value	RR (95% CI)
Failure patterns	0.997	–
Initial treatment	0.287	–
RFI time	0.042	0.743 (0.559–0.989)
Chemotherapy	0.001	0.580 (0.420–0.802)
Salvage radiation dose	0.379	–
Esophageal stenosis	0.045	1.319 (1.007–1.729)
Fat space between tumor and adjacent tissue disappeared	0.158	–
Hb level	0.155	–

*RR* relative risk, *CI* confidence interval

### Toxicity

EP was the most common complication in all patients received re-RT (21.4%, 54/252), 40 patients occurred in initial CRT group and 14 patients in surgery group (Table 5). 12 patients were treated with stenting and 14 patients were treated with bouginage before re-RT, and 8 patients developed EP after endoscopic treatment, of whom 6 patients received stenting and 2 patients had bouginage treatment. Radiation-induced pneumonitis was observed in 32 patients (12.8%), 10 patient (4.0%) experienced grade 3 radiation pneumonitis and 3 patients died due to radiation pneumonitis. Esophagorrhagia was noted in 23 patients (9.1%), which was more frequent in patients who received surgery initially (12.9%, 11/85). 17 patients in CRT group and 6 patients in surgery group died of esophagorrhagia, respectively.

**Table 5:** Comparison of baseline characteristics between the patients with EP ( +) and EP (−) after re-RT

Variables	EP (−)	EP ( + )	*p*
Re-RT age (years) (n, %)			0.693
≤ 60	68 (34.3)	17 (31.5)	
> 60	130 (65.7)	37 (68.5)	
Gender (n, %)			0.955
Male	162 (81.8)	44 (81.5)	
Female	36 (18.2)	10 (18.5)	
Smoking (n, %)			0.965
Yes	96 (48.5)	26 (48.1)	
No	102 (51.5)	28 (51.9)	
Alcohol consumption (n, %)			0.206
Yes	80 (40.4)	27 (50)	
No	118 (59.6)	27 (50)	
Primary tumor location (n, %)			
Upper	53 (26.8)	19 (35.2)	0.225
Middle and lower thoracic	145 (73.2)	35 (64.8)	
Length, cm (n, %)			0.872
≤ 4	121 (61.1)	31 (57.4)	
4 < to ≤ 6	55 (27.8)	16 (29.6)	
> 6	22 (11.1)	7 (13.0)	
Ulcerative type (n, %)			0.546
Yes	109 (55.1)	25 (46.3)	
No	89 (44.9)	29 (53.7)	
Tumor differentiation (n, %)			0.882
Higher	29 (14.6)	7 (13.0)	
Middle	113 (57.1)	30 (55.6)	
Lower	56 (28.3)	17 (31.4)	
T stage (n, %)			0.962
T1–2	63 (31.8)	17 (31.5)	
T3–4	135 (61.2)	37 (68.5)	
Initial clinical/pathological stage (n, %)			0.775
I–II	91 (46.0)	26 (48.1)	
III–Iva	107 (54)	28 (51.9)	
Initial treatment (n, %)			0.171
RT	127 (64.1)	40 (74.1)	
Surgery + RT	71 (35.9)	14 (25.9)	
Pattern of recurrence (n, %)			0.010
Regional lymph node recurrence only	90 (45.4)	15 (27.8)	
Local failure	73 (36.9)	20 (37.0)	
Both	35 (17.7)	19 (35.2)	
Recurrence-free interval (months) (n, %)			0.025
≤ 12	53 (36.8)	23 (42.6)	
> 12	145 (73.2)	31 (57.4)	
Re-RT dose (Gy) (n, %), BED_10_			0.442
≤ 60	102 (51.5)	31 (53.7)	
> 60	96 (48.5)	23 (46.3)	
Concurrent chemoradiotherapy (n, %)			0.894
Yes	86 (43.4)	24 (44.4)	
No	112 (56.6)	30 (55.6)	
Total radiation dose (Gy) (n, %), BED_10_			0.334
≤ 131.5	99 (50.0)	31 (57.4)	
> 131.5	99 (50.0)	23 (42.6)	
Esophageal stenosis (n, %)			0.000
Yes	68 (34.3)	34 (63.0)	
No	130 (65.7)	20 (37.0)	
Fat space between tumor and adjacent tissue disappeared (n, %)			0.000
Yes	37 (18.7)	27 (50.0)	
No	161 (81.3)	27 (50.0)	
Pain in the chest or/and back (n, %)			0.175
Yes	17 (8.6)	8 (14.8)	
No	181 (91.4)	46 (85.2)	
Median BMI (n, %)			0.102
≤ 20	63 (31.8)	11 (20.4)	
> 20	135 (68.2)	43 (79.6)	
Hemoglobin (n, %)			0.141
≤ 12	70 (35.4)	25 (46.3)	
> 12	128 (64.6)	29 (53.7)	
Albumin (n, %)			0.447
≤ 40	66 (33.3)	21 (38.9)	
> 40	132 (66.7)	33 (61.1)	
White blood cells, × 10^9^/L	5.776 ± 2.285	6.520 ± 3.103	0.059
Neutrophil, × 109/L	4.379 ± 2.172	5.013 ± 2.936	0.090

### Risk factors for EP

We evaluated the relationship between EP and clinicopathological features (Table 6). Univariate analysis revealed that pattern of recurrence, RFI, esophageal stenosis and fat space between tumor and adjacent tissue disappeared were potential risk factors for EP after re-RT. We then used the forward logical regression method to perform a multivariant analysis for the post re-RT EP risk factors. We found that the RFI ≤ 12 months, esophageal stenosis and fat space between tumor and adjacent tissue disappeared were independent risk factors for the development of EP after re-RT.

**Table 6:** Univariate and multivariate logistic analysis of risk factors of EP after re-RT

Variables	Univariate analysis			Multivariate analysis		
	HR	95% CI	*p*	HR	95% CI	*p*
Re-RT age (years)	1.114	0.610–2.036	0.725			
Gender (n, %)	0.978	0.450–2.124	0.955			
Smoking (n, %)	0.987	0.540–1.802	0.965			
Alcohol consumption (n, %)	1.444	0.790–2.642	0.233			
Primary tumor location (n, %)	0.673	0.355–1.278	0.227			
Length, cm (n, %)	1.120	0.732–1.714	0.602			
Macroscopic tumor type	1.210	0.881–1.662	0.240			
Tumor differentiation (n, %)	1.127	0.703–1.805	0.620			
T stage (n, %)	1.049	0.693–1.587	0.822			
N stage (n, %)	0.908	0.597–1.382	0.652			
Initial clinical/pathological stage (n, %)	1.113	0.738–1.679	0.610			
Initial treatment	1.597	0.814–3.135	0.173			
Pattern of recurrence (n, %)	1.360	0.911–2.031	0.133			
Recurrence-free interval (months)	0.493	0.264–0.920	0.026	0.450	0.229–0.884	0.021
Re-RT dose (Gy)	0.788	0.430–1.447	0.443			
Concurrent chemoradiotherapy	1.042	0.568–1.910	0.894			
Total radiation dose (Gy)	0.742	0.404–1.361	0.335			
Esophageal stenosis	3.250	1.739–6.074	0.000	2.665	1.359–5.225	0.004
Pain in the chest or/and back	2	0.752–4.557	0.180			
Fat space between tumor and adjacent tissue disappeared	4.351	2.290–8.269	0.000	3.347	1.702–6.581	0.000
BMI	1.824	0.882–3.773	0.105			
Hemoglobin (g/L)	0.634	0.345–1.166	0.143			
Albumin (g/L)	0.786	0.422–1.463	0.447			
White blood cells, × 10^9^/L	1.133	1.1011–1.270	0.032			
Neutrophil, × 10^9^/L	1.121	0.995–1.263	0.059			

*HR* hazard ratio, *CI* confidence interval

## Discussion

LR of ESCC can be a devastating condition, because of the patients should bear obstruction, dysphagia, pain, infection, bleeding, nausea and vomiting with large impact on health-related quality of life. In the whole population, the recurrence rate of regional LN is slightly higher than PF (42.9% vs. 38.1%). PF recurrence rate was lower than previous study [17, 20], just because of patients who underwent RT after surgery initially and received re-RT were included in the analysis. Regional LN and PF were the most common failure pattern in the surgery group (70.6%) and the radical RT group (50.3%), respectively. These patients are usually in good physical condition, and they are expected to get better survival by taking reasonable salvage treatments.

But the role of salvage treatments in patients with LR in the primary tumor bed after RT is controversial [21]. Re-irradiation has been successfully used in many recurrent tumors of various sites with the development of radiotherapy techniques, such as head and neck cancer [7, 22], high grade glioma [23], lung cancer [24], intracranial germ-cell tumors [25], rectal cancer [26, 27] and paediatric tumor [28] with encouraging results. Several small size retrospective studies reported the outcome of re-RT of LR for ESCC patients received RT [14, 17, 29]. The only prospective study reported that re-RT for oligo-recurrence in lymph nodes from esophageal cancer treated by definitive RT or by surgery with additional RT might be acceptable but unsatisfactory [15]. In our present study, we found that there was a significant increase in OS for patients who received re-RT. The 5-years OS rate was 32.1% in salvage radiotherapy patients, and the median survival time was 39.0 months, which is longer than those reported in other studies [13, 14]. The median ARS was 13.0 months (range 3–168 months), which was similar to the results of previous studies [12–17, 29]. Therefore, further research is needed to improve the survival time after recurrence.

The factors that influence the efficacy of re-RT are also of interest to researchers. In the present study, we also found failure pattern was associated with ARS, and LN recurrence had better survival than PF and/or combined with LN (*p* = 0.026), which is consistent with Hong et al. [17] reports. Although previous study had not found significant difference in the effect of RFI on prognosis [14], but we found that patients with RFI > 12 months had better outcomes (14 vs. 11 months, *p* = 0.037) through univariate analysis and Cox regression analysis. This might be attributed to differences in the baseline differences in the population and tumor cells that do not respond to the treatment for early recurrences [30].

We also found that the failure pattern was associated with ARS after re-RT in univariant analysis. Hong et al. [17] found that the median survival time (MST) in the LN group was 23 months, whereas the MST in the PF group was 9 months (*p* = 0.004). The LN group had better ARS than the PF group with/without LN (*p* = 0.026) in our study, this may be related to the nutritional status of patients with PF and their poorer tolerance to treatment. Minsky et al. [31] confirmed that higher radiation dose did not increase the survival or improved the local/regional control for esophageal cancer in trial INT 0123 and used 50.4 Gy as a standard irradiation dose. We also found that the ARS of the patients received total RT dose with BED > 131.5 Gy were similar to patients received a total dose with BED ≤ 131.5 Gy (*p* = 0.545). Meanwhile, salvage radiation dose did not affect ARS in our present study (*p* = 0.326). This may be related to the fact that the basic conditions of the patients are different during the re-RT and the salvage radiation dose is difficult to be fixed. It also partially suggests that increasing radiation dose alone may not improve survival for LR ESCC patients.

It is well known that CT can improve the sensitivity of radiotherapy and improve the therapeutic effect. But in Hong et al. study, no statistical difference in ARS was observed between the groups treated with re-RT alone and re-RT combined with CT (*p* = 0.70) [17]. In our present study, we found that patients received re-RT combined with CT got better ARS than patients who were not (*p* = 0.001). We recommended CT for the patients who received re-RT in good physical condition. Patients with RFI > 12 months had better ARS than RFI ≤ 12 months, this may be related to the different sensitivity of tumor to RT, and patients with longer RFI may be more sensitive to RT. Our findings are in agreement with those reported by previous studies [14, 30]. Esophageal stenosis predicts nutritional status and poorer tolerance to treatment, which affects the patient's prognosis. Previous report found that esophageal stenosis was a predictor of poor median overall and recurrence-free survival in patients with oesophageal cancer [32]. We found that esophageal stenosis also affected the prognosis of patients receiving re-RT. Therefore, further assessment of these risk factors will contribute to a more accurate assessment of the patient's prognosis.

In addition to improve the prognosis, it is also important to predict and prevent adverse effects associated with re-RT. Zhou et al. [14] reported that the EP was observed in 11 cases (20.0%) and in 8 cases (13.6%) in the re-RT and non-salvage re-RT group, respectively (*p* = 0.357). Chen et al. [13] showed that esophagotracheal fistula in 5 patients and esophageal perforation in 2 patients were identified in the re-RT group (n = 36). In the current study, EP occurred in 21.4%, which is similar to pervious reports.

It is important to predict adverse effects associated with re-irradiation. Patients with RFI ≤ 12 months, esophageal stenosis and fat space between tumor and adjacent tissue disappeared had a higher risk of EP through our present analysis. There is no prediction of risk factors for EP caused by re-RT in the past, but the risk factors were similar to patients who received radiotherapy for the first time [18, 33]. Other risk factors for EP in patients undergoing RT for the first time have also been reported [34–36], further study on the risk of EP in re-RT is expected to be included in analysis. Patients with esophageal stenosis might be treated with stenting/bouginage, which is a risk factor for EP especially in patients who underwent RT [37]. In our present study, 30.8% (8/26) of patients received re-RT after endoscopic treatment developed EP, which was much higher than the overall incidence. Therefore, patients received stenting/bouginage should be closely observed during re-RT and nasal feeding diet or intravenous nutrition during re-RT are also optional treatment strategies. RP is another complication which should be concerned in re-RT. The incidence of grade 3 RP was 12.5% for re-RT group in our study, which is similar to previous study [14, 17]. Bleeding rate was very low in re-RT population [13], which was higher in our study may be because of thoracic stomach increases the risk of esophagorrhagia. Although the incidence of complications in re-RT is acceptable according to previous reports and the findings of our study, we should pay more attention to these patients. Further studies are required to assess the risk factors for toxicities through re-RT.

The present study has several limitations. First, the treatment time span was longer, resulting in poor consistency of treatment. Second, we were unable to analyze how the re-RT dose determined, which is probably related to the patient's nutritional status and tolerance to treatment.

## Conclusion

Re-RT was feasible for LR ESCC patients after RT initially, it was also proved effective and safe to receive re-RT for initial surgery patients. Combined with chemotherapy and RFI time > 12 months were better prognostic factors for ARS, and patients with esophageal stenosis may have a poor prognosis. Patients with RFI ≤ 12 months, esophageal stenosis and fat space between tumor and adjacent tissue disappeared should be paid more attention, because of these patients are at significantly increased risk of EP.

## Data Availability

Data are available from the author when needed.

## Abbreviations

LR: Locoregional recurrence
CRT: Chemoradiotherapy
RT: Radiotherapy
ESCC: Esophageal squamous cell carcinoma
Re-RT: Re-irradiation
PF: Primary failure
LN: Lymph node recurrence
GTV: Gross tumor volume
CTV: Clinical target volume
PTV: Planning target volume
CT: Chemotherapy
OS: Overall survival
ARS: After-recurrence survival
RFI: Recurrence-free interval
EP: Esophageal fistula/perforation
RP: Radiation pneumonitis
MST: Median survival time
